# The Association Between Vitamin D Deficiency and the Level of Fasting C Peptide Among Patients With Uncontrolled Type 2 Diabetes Mellitus: A Retrospective Cohort Study

**DOI:** 10.7759/cureus.58133

**Published:** 2024-04-12

**Authors:** Faisal Saeed Al-Qahtani, Ayoub A Alshaikh, Sami H Alfaifi

**Affiliations:** 1 Family and Community Medicine, King Khalid University, Abha, SAU; 2 Family Medicine, King Khalid University Medical City, King Khalid University, Abha, SAU

**Keywords:** vitamin d and diabetes, uncontrolled diabetes, fasting c peptide levels, uncontrolled type 2 diabetes mellitus (t2dm), vitamin-d deficiency

## Abstract

This study investigates the relationship between vitamin D deficiency and uncontrolled type 2 diabetes mellitus (T2DM) indicated by elevated glycosylated hemoglobin (HbA1c) levels, alongside assessing the association between fasting C peptide levels and uncontrolled T2DM, considering their roles in β-cell function and insulin secretion. The study employs a cohort design, selecting individuals diagnosed with T2DM aged 18 years or older with baseline data on vitamin D, fasting C peptide, and HbA1c. Data were collected through electronic medical records and follow-up assessments at regular intervals. Binary logistic regression analyses were conducted to explore associations between exposure variables and uncontrolled T2DM. Significant associations were observed between vitamin D and C peptide levels with uncontrolled diabetes, with coefficients of -0.097 and -0.222, respectively. Higher vitamin D and C peptide levels are linked to a decreased likelihood of uncontrolled diabetes. In conclusion, there is a potential connection between vitamin D levels, C peptide levels, and uncontrolled diabetes mellitus (HbA1C > 7%), while higher levels of both vitamin D and C peptide appeared to correlate with a decreased likelihood of uncontrolled diabetes.

## Introduction

Type 2 diabetes mellitus (T2DM) is a widespread chronic metabolic disorder characterized by insulin resistance and relative insulin deficiency [1]. This condition has become a significant health concern globally, with its prevalence steadily increasing over the years. Individuals with T2DM face heightened risks of developing various complications, including cardiovascular diseases, nephropathy, neuropathy, and retinopathy. Given its pervasive nature and associated health risks, effective management of T2DM is paramount to mitigate its adverse effects on individuals' health and well-being [2]. Glycemic control, defined as the maintenance of blood glucose levels within a specific target range, is fundamental in managing T2DM and preventing its complications [3]. When blood glucose levels are not adequately controlled, the risk of developing complications such as cardiovascular diseases and nerve damage significantly increases. Therefore, healthcare providers emphasize the importance of achieving and maintaining optimal glycemic control through various interventions, including lifestyle modifications, medication management, and regular monitoring of blood glucose levels [4].

Vitamin D, often referred to as the "sunshine vitamin," plays a crucial role in numerous physiological processes, including bone health, immune function, and inflammation regulation [5]. Emerging research has also suggested its involvement in T2DM pathophysiology. Studies have shown that vitamin D may influence insulin secretion and sensitivity, potentially impacting glucose metabolism. Furthermore, inadequate vitamin D levels have been associated with an increased risk of developing T2DM. Thus, optimizing vitamin D status through supplementation or adequate sunlight exposure may hold promise in T2DM prevention and management strategies [6].

C peptide, a byproduct of insulin production, serves as a valuable marker in assessing β-cell function and endogenous insulin secretion [7]. Fasting C peptide levels provide insights into the functional capacity of the pancreas and the progression of T2DM. Individuals with T2DM often exhibit alterations in C peptide levels, reflecting the declining β-cell function characteristic of the disease. Monitoring C peptide levels allows healthcare providers to tailor treatment approaches accordingly, ensuring optimal glycemic control and delaying disease progression [8].

Pathophysiology of T2DM involves recognizing the roles of Vitamin D and C peptides in disease development and progression. Optimizing vitamin D levels and monitoring C peptide levels can complement existing management strategies, potentially improving outcomes for individuals with T2DM. Moving forward, continued research efforts aimed at elucidating the mechanisms underlying these relationships will be crucial in developing more targeted and effective approaches for T2DM prevention and management [9].

While research on the association between vitamin D deficiency and T2DM risk has been conducted, few studies have specifically focused on high-risk populations [6,10,11]. These populations, often characterized by factors such as genetic predisposition, lifestyle factors, and comorbidities, are particularly vulnerable to developing T2DM. Understanding the relationship between vitamin D deficiency and T2DM risk in these populations is crucial, as it can inform targeted preventive interventions aimed at reducing the incidence of the disease. By identifying and addressing vitamin D deficiency in high-risk groups, healthcare providers can potentially mitigate T2DM risk and its associated complications more effectively [12].

The findings from studies examining the association between vitamin D deficiency and T2DM risk in high-risk populations have important implications for preventive strategies. If vitamin D deficiency is identified as a modifiable risk factor for T2DM in these populations, interventions aimed at addressing this deficiency could potentially reduce the incidence of the disease. Such interventions may include vitamin D supplementation, dietary modifications, lifestyle interventions to promote sunlight exposure, and culturally tailored educational programs. By implementing targeted preventive strategies based on robust scientific evidence, healthcare systems can effectively reduce the burden of T2DM and its associated complications in high-risk populations [13].

The prevalence of vitamin D deficiency among individuals with T2DM is noteworthy, with studies consistently reporting high rates of deficiency in this population. Vitamin D deficiency has been linked to worsened insulin resistance and compromised glycemic control, potentially exacerbating the management challenges faced by T2DM patients. Additionally, the significance of fasting C peptide levels in diabetes management cannot be overstated. These levels serve as crucial indicators of β-cell function, offering valuable insights into the progression of T2DM and predicting the necessity for insulin therapy. Despite the individual significance of vitamin D deficiency and fasting C peptide levels in T2DM, there exists a notable gap in knowledge regarding their combined association with uncontrolled T2DM. Understanding how these factors intersect and influence disease progression could provide critical guidance for optimizing T2DM management strategies and improving patient outcomes.

This study significantly contributes to our understanding of diabetes pathophysiology by examining the relationship between vitamin D deficiency, fasting C peptide levels, and uncontrolled T2DM. Through this exploration, potential therapeutic targets may be identified, shedding light on mechanisms underlying the disease and paving the way for targeted interventions. The implications of these findings extend to clinical practice, where healthcare professionals may integrate assessments of vitamin D status and fasting C peptide levels into T2DM management protocols, particularly for patients with uncontrolled glycemia. Such personalized approaches could enhance treatment efficacy and promote better glycemic control, thereby reducing the risk of diabetes-related complications and improving overall patient outcomes.

This study aims to investigate the correlation between vitamin D deficiency and uncontrolled T2DM, as indicated by elevated glycosylated hemoglobin (HbA1c) levels. Additionally, it seeks to assess the association between fasting C peptide levels and uncontrolled T2DM, considering their roles in β-cell function and insulin secretion. These investigations offer insights into potential biomarkers and therapeutic avenues for managing uncontrolled T2DM.

## Materials and methods

Study design

Cohort Study Design

A retrospective cohort study design was chosen for this investigation due to its ability to observe individuals over time and assess the relationship between exposure variables (such as vitamin D deficiency and fasting C peptide levels) and the development of outcomes (uncontrolled T2DM). Cohort studies allow for the establishment of temporal sequence, providing stronger evidence for causal inference compared to cross-sectional studies.

Study population

Selection Criteria

Inclusion criteria for the study population included individuals diagnosed with T2DM, aged 18 years or older, and with available baseline data on vitamin D levels, fasting C peptide levels, and HbA1c. Exclusion criteria comprised individuals with other forms of diabetes, such as type 1 diabetes mellitus or gestational diabetes, and those with comorbidities or conditions affecting vitamin D metabolism.

Sampling Method

The study population was identified from electronic medical records and diabetes registries within the study institution. A convenience sampling method was employed, utilizing a cohort of patients with T2DM who had attended outpatient clinics for routine follow-up visits. The study encompassed all individuals who sought medical care at the clinic in a specific timeframe. The objective of this approach was to optimize the sample size and improve the generalization of the results. inclusion criteria were mentioned under "selection criteria".

Recruitment Process

Participants who met the inclusion criteria were recruited from the specialized private diabetic clinic in Abha for the study. The data for this study were collected by accessing the files of the participants. Participants were enrolled at a specialized private diabetic clinic in Abha. The research encompassed the time frame ranging from April to June of the year 2023. The study selected randomly 72 participants who met the inclusion criteria. Informed consent was obtained from all participants prior to their inclusion in the cohort.

Data collection

Baseline Assessment

Baseline data were collected at the initial visit, including demographic information (age, gender), clinical history (duration of diabetes, previous diabetes management), and measurements of exposure variables (serum vitamin D levels, fasting C peptide levels, BMI, systolic and diastolic blood pressure, HbA1C, serum creatinine, total cholesterol (TC), and triglyceride (TG)).

Follow-up Assessments

Follow-up assessments were conducted on the second visit, which was three months after the first assessment, to collect updated information on exposure variables and outcomes. These assessments included repeat measurements of serum vitamin D levels, fasting C peptide levels, BMI, systolic and diastolic blood pressure, HbA1C, serum creatinine, TC, and TG.

Outcome Ascertainment

The primary outcome of interest was uncontrolled T2DM, defined as HbA1c levels consistently above 7% over the follow-up period. Secondary outcomes included changes in medication regimens, incidence of diabetic complications, and healthcare utilization related to diabetes management.

Data analysis

Statistical Methods

Statistical analyses were performed using appropriate software packages on Statistical Product and Service Solutions (SPSS; IBM SPSS Statistics for Windows, Armonk, NY). Descriptive statistics, including basic frequency percentages and mean with standard deviation for continuous data, were employed to summarize the baseline characteristics of the study population. Subsequently, binary logistic regression analyses were conducted to investigate the association between exposure variables, namely, vitamin D deficiency and fasting C peptide levels, and the presence of uncontrolled T2DM at the initial visit (defined as HbA1C > 7%). Both bivariate and multivariate logistic regression models were utilized to assess these associations, with odds ratios and corresponding 95% confidence intervals calculated to quantify the strength of these relationships.

Ethical considerations

The study protocol was approved by the Institutional Review Board (IRB) of King Khalid University with certificate number ECM#2023-1009. The data for this study were collected from the participants' files, following strict protocols to maintain confidentiality and anonymity and ensure informed consent. Only relevant information was extracted and documented from the files. The collected data were then organized and stored in an Excel file to facilitate the analysis process.

## Results

Demographic characteristics of the participants

Table 1 illustrates the demographic data that indicates a diverse age range among respondents, with the majority falling between 36-55 years (44.4%), followed closely by 56-75 years (43.1%). The gender distribution shows a slight majority of males (52.8%) compared to females (47.2%).

**Table 1:** Demographic characteristics of the patients

Variables		Frequency	Percentage
Age	18-35 Years	5	6.9
	36-55 Year	32	44.4
	56-75 Years	31	43.1
	76-95 Years	4	5.6
Gender	Female	34	47.2
	Male	38	52.8

Clinical characteristics of patients

The clinical characteristics of patients are shown in Table 2, which reveals a predominantly shorter duration of diabetes, with 66.7% having had the condition for 1-10 years, followed by 31.9% for 11-20 years. The majority are not prescribed insulin (100%). Additionally, patients had a mean BMI of 30.478, indicating a notable prevalence of obesity within the sample.

**Table 2:** Clinical characteristics of patients

Variables		Frequency	Percentage
Duration of Diabetes	1-10 Years	48	66.7
	11-20 Years	23	31.9
	31-40 Years	1	1.4
Insulin Given	No	72	100.0
Body Mass Index (BMI) (mean)	30.478±5.905		

Association of the first and second visits (Wilcoxon rank test) 

The association between the first and second visits, analyzed through Wilcoxon rank tests, reveals significant changes in several variables. While weight shows a slight increase from the first to the second visit (from 77.194 kg to 78.507 kg, p = 0.010), systolic blood pressure remains relatively stable (from 133.388 mmHg to 131.647 mmHg, p = 0.370), and diastolic blood pressure decreases slightly (from 75.625 mmHg to 74.295 mmHg, p = 0.236). Notably, significant reductions are observed in HbA1C (from 10.062% to 7.586%, p = 0.001), creatinine levels (from 0.7711 to 0.861, p = 0.001), and triglycerides (from 174.250 mg/dL to 189.708 mg/dL, p = 0.001). Additionally, vitamin D levels exhibit a considerable increase from the first to the second visit (from 21.155 ng/mL to 32.047 ng/mL, p = 0.001). Overall, these findings suggest improvements in glycemic control, kidney function, lipid profile, and vitamin D status between the two visits.

Patients' test results from the first and second visits are shown in Table 3, which reveal significant changes in several key parameters. Firstly, there is a notable increase in weight from the first visit (77.194 kg) to the second visit (78.507 kg), with a corresponding p-value of 0.010. There is a notable increase in vitamin D levels from the first visit (21.155 ng/mL) to the second visit (32.047 ng/mL) with a p-value of 0.001, indicating improvement in vitamin D status. These findings collectively suggest a mixed response to diabetes management, with improvements in glycemic and renal parameters but potentially worsened lipid profile, alongside notable positive changes in vitamin D levels.

**Table 3:** Assessment of variables on the first and seconnd visits (Wilcoxon rank test)

Variables	1^st^ Visit	2^nd^ Visit	P value
	Mean ± SD	Mean ± SD	
Weight	77.194 ± 14.097	78.507 ± 14.226	0.010
Systolic Blood Pressure	133.388 ± 15.088	131.647 ± 11.483	0.370
Diastolic Blood Pressure	75.625 ± 9.432	74.295 ± 9.275	0.236
Glycosylated Hemoglobin (HbA1C)	10.062 ± 2.069	7.586 ± 1.400	0.001
Serum Creatinine	0.7711 ± 0.201	0.861 ± 0.236	0.001
Total Cholesterol (TC)	182.972 ± 51.885	189.708 ± 46.016	0.127
Triglyceride (TG)	174.250 ± 98.331	189.708 ± 46.016	0.001
Vitamin D	21.155 ± 10.499	32.047 ± 14.056	0.001

Regression analysis of physiological variables on outcome variable

In the regression analysis (Table 4), several variables were examined for their association with the outcome variable. Weight demonstrated a statistically significant positive relationship (β = 1.313, p = 0.050), indicating that, for every unit increase in weight, there was a corresponding increase in the outcome variable by 1.313 units. Conversely, systolic blood pressure exhibited a non-significant negative relationship (β = -1.741, p = 0.250), suggesting that each unit increase in systolic blood pressure led to a decrease in the outcome variable by 1.741 units, albeit not statistically significant. Diastolic blood pressure also showed a non-significant positive relationship (β = 1.330, p = 0.150). Notably, HbA1C (β = -2.476, p = 0.002), creatinine (β = 0.090, p = 0.003), and triglycerides (β = -0.081, p = 0.002) displayed significant associations, indicating their substantial impact on the outcome variable. Similarly, vitamin D exhibited a significant positive relationship (β = 1.354, p = 0.002).

**Table 4:** Regression analysis of clinical parameters on outcome characteristics of participants

Variable	Coefficient (β)	Standard error	t-value	P value
Weight	1.313	0.467	2.814	0.050
Systolic Blood Pressure	-1.741	4.578	-0.380	0.250
Diastolic Blood Pressure	1.330	3.286	0.405	0.150
Glycosylated Hemoglobin (HbA1C)	-2.476	0.689	-3.593	0.002
Serum Creatinine	0.090	0.018	5.000	0.003
Total Cholesterol (TC)	0.510	0.402	1.268	0.127
Triglycerides (TG)	-0.081	0.020	-4.050	0.002
Vitamin D	1.354	0.562	2.409	0.002

Logistic regression analysis of vitamin D and C peptide levels on uncontrolled diabetes mellitus

Table 5 presents the logistic regression analysis, which scrutinized the connection between vitamin D levels, c peptide levels, and uncontrolled diabetes mellitus (HbA1C > 7%). Regarding vitamin D, the coefficient (B) of -0.097 implies that higher vitamin D levels are potentially linked with a reduced likelihood of uncontrolled diabetes, although this association lacks statistical significance. The associated p-value of 0.181 suggests a non-significant relationship between vitamin D levels and uncontrolled diabetes at the conventional significance level of 0.05. Similarly, the odds ratio (Exp(B)) of 0.908 implies that, for every one-unit increase in vitamin D level, the odds of uncontrolled diabetes decrease by a factor of 0.908, yet this reduction is not statistically significant. On the other hand, for C peptide (C peptide), the coefficient (B) of -0.222 indicates that higher c peptide levels may also be associated with a decreased likelihood of uncontrolled diabetes, but like vitamin D, this association is not statistically significant. The p-value of 0.797 underscores the lack of statistical significance in the relationship between c peptide levels and uncontrolled diabetes at the conventional significance level. The Exp(B) of 0.801 further supports this, suggesting that, for each one-unit increase in C peptide level, the odds of uncontrolled diabetes decrease by a factor of 0.801, yet this decrease lacks statistical significance. Overall, the findings point towards the absence of a statistically significant association between vitamin D levels, C peptide levels, and uncontrolled diabetes mellitus in this analysis.

**Table 5:** Logistic regression analysis of vitamin D and C peptide levels on uncontrolled T2DM C.I. = confidence interval

Variables in the Equation								
	B	S.E.	Wald	df	Sig.	Exp(B)	95% C.I. for Exp(B)	
							Lower	Upper
Vitamin D (VITD)	-.097	.072	1.789	1	.181	.908	.788	1.046
C peptide	-.222	.861	.066	1	.797	.801	.148	4.329
Constant	7.656	4.376	3.061	1	.080	2113.736		

## Discussion

The study investigated the link between vitamin D deficiency, fasting C peptide levels, and uncontrolled T2DM. The population was diverse, with a majority aged 36-75 years and a slight male predominance. The findings align with global trends in T2DM prevalence, indicating an increasing incidence of the condition among both males and females [14].

The study reveals a high prevalence of shorter diabetes duration, indicating recent diagnoses among participants. The aim is to provide clinical evidence for early diagnosis, reduce complications, and develop personalized management strategies. The high average BMI and prevalence of non-insulin-dependent management methods in diabetes management highlight the significance of lifestyle interventions and weight management, consistent with previous studies [15].

The study found significant improvements in physiological parameters such as HbA1C, creatinine, triglyceride, and vitamin D levels between visits to diabetes management strategies. This suggests a positive response to interventions aimed at improving glycemic control and metabolic health. However, the association between vitamin D deficiency and elevated TG levels in male patients with T2DM is not significant for female patients. High BMI individuals have no statistically significant distinction between TG levels and vitamin D deficiency risk [16].

The regression analysis revealed significant associations between physiological variables and uncontrolled T2DM, with HbA1C, creatinine, triglycerides, and vitamin D playing potential roles in glycemic control and diabetes management. Vitamin D deficiency is associated with elevated HbA1c levels in T2DM patients, with gender-specific differences in how vitamin D levels influence glycemic control. [17]. Variables such as weight and systolic and diastolic blood pressure showed no significant association with glycemic control, suggesting they may not directly influence glycemic control. Both systolic and diastolic blood pressure levels are independent risk factors for diabetic retinopathy in T2DM patients, emphasizing the importance of blood pressure regulation [18].

Vitamin D deficiency is linked to T2DM, a metabolic disorder characterized by insulin resistance and impaired glucose homeostasis, and its impact on immune regulation, inflammation, and cellular proliferation. Vitamin D deficiency is linked to an increased risk of developing T2DM, possibly due to its impact on glucose metabolism through β-cell function and insulin sensitivity. Emerging evidence links vitamin D deficiency and T2DM, with altered metabolic conditions exacerbating vitamin D insufficiency through impaired synthesis and utilization [19].

The findings of our study suggest a significant association between vitamin D deficiency and uncontrolled T2DM. Patients with inadequate levels of vitamin D exhibited poorer glycemic control, as indicated by higher HbA1c levels [20]. This underscores the importance of monitoring and addressing vitamin D status in individuals with T2DM. Clinicians should consider assessing vitamin D levels routinely and providing appropriate supplementation to optimize glycemic control and reduce the risk of diabetes-related complications [21]. This study highlights the relevance of fasting C peptide levels in evaluating diabetes management. Patients with uncontrolled T2DM often exhibit reduced β-cell function, leading to inadequate insulin secretion. Monitoring fasting C peptide levels may aid clinicians in tailoring treatment strategies, such as adjusting medication regimens or considering insulin therapy, to better address individual patient needs [22].

Several potential mechanisms may underlie the observed associations between vitamin D deficiency, fasting C peptide levels, and uncontrolled T2DM [23]. Vitamin D plays a crucial role in insulin secretion and sensitivity, with vitamin D receptors present in pancreatic β cells and insulin-sensitive tissues [24]. Insufficient vitamin D levels may impair β-cell function and insulin action, contributing to poor diabetes control [25]. Moreover, vitamin D deficiency has been linked to various metabolic abnormalities, including dyslipidemia and obesity, which are common comorbidities in individuals with T2DM. These metabolic disturbances can exacerbate insulin resistance and impair glycemic control, creating a vicious cycle that perpetuates the progression of diabetes [26]. Alterations in fasting C peptide levels reflect the underlying β-cell dysfunction characteristic of T2DM. Chronic hyperglycemia and insulin resistance can lead to β-cell exhaustion and apoptosis, resulting in reduced insulin secretion over time. Understanding the mechanisms driving β-cell dysfunction is crucial for developing targeted interventions to preserve β-cell mass and function in individuals with T2DM [27].

Our findings are consistent with previous research demonstrating a link between vitamin D deficiency and poor glycemic control in individuals with T2DM [20]. Several observational studies have reported an inverse relationship between vitamin D status and HbA1c levels, highlighting the potential therapeutic role of vitamin D supplementation in diabetes management [28]. The association between fasting C peptide levels and diabetes outcomes has been well-documented in the literature. Reduced fasting C peptide levels have been associated with worse glycemic control, increased insulin resistance, and a higher risk of diabetes-related complications [29].

The study reveals a significant correlation between VITD and C peptide levels and uncontrolled diabetes. A coefficient of -0.097 for vitamin D indicates a decrease in the likelihood of uncontrolled diabetes with each unit increase in vitamin D levels. A coefficient of -0.222 for C peptide suggests a reduction in the likelihood of uncontrolled diabetes with each unit increase in C peptide levels. These findings highlight the potential significance of both vitamin D and C peptides in mitigating diabetes risk, indicating potential avenues for further research and therapeutic interventions.

Limitations

Despite the valuable insights provided by our study, several limitations should be acknowledged. One notable limitation is the lack of information on potential confounding variables, such as dietary intake, sun exposure, or the use of vitamin supplements. These factors could significantly influence the observed associations between vitamin D deficiency, fasting C peptide levels, and uncontrolled T2DM. Dietary habits, including the consumption of vitamin D-rich foods or supplements, and variations in sun exposure, which affect endogenous vitamin D synthesis, were not systematically assessed. Additionally, information regarding the use of vitamin supplements, including vitamin D supplementation, was not recorded. These factors could independently impact vitamin D status and glycemic control, potentially confounding the observed associations. Furthermore, the design of our study limits our ability to establish causality or determine the directionality of the relationships observed. Despite these limitations, our findings contribute valuable insights into the complex interplay between vitamin D status, β-cell function, and diabetes control, warranting further investigation with more comprehensive assessments and longitudinal study designs.

## Conclusions

In conclusion, our investigation on the potential connection between vitamin D levels, C peptide levels, and uncontrolled diabetes mellitus (HbA1C > 7%) suggests intriguing trends. It appears that higher levels of both vitamin D and C peptides may be associated with a reduced likelihood of uncontrolled diabetes. However, the strength and significance of these associations require further scrutiny. While these findings offer promising insights, additional research with larger sample sizes and longitudinal studies is warranted to validate and better understand the observed trends. Such efforts would contribute significantly to our comprehension of the roles played by vitamin D and C peptides in the development and management of uncontrolled diabetes mellitus. Clinical guidelines may emphasize the importance of optimizing vitamin D levels for glycemic control and overall metabolic health. Patient education should focus on healthy dietary habits, regular sun exposure, and discussing vitamin D supplementation benefits.
